# Type II Lip Pattern among Medical Students in a Medical College: A Descriptive Cross-sectional Study

**DOI:** 10.31729/jnma.7690

**Published:** 2022-09-30

**Authors:** Barshika Katwal, Deepika Karki, Asim Shrestha

**Affiliations:** 1 Department of Forensic Medicine, Nepal Medical College and Teaching Hospital, Jorpati, Kathmandu, Nepal; 2 Nepal Medical College and Teaching Hospital, Jorpati, Kathmandu, Nepal

**Keywords:** *forensic dentistry*, *lip*, *pattern*, *personal identification system*

## Abstract

**Introduction:**

The study of human identification is of great value in forensic medicine. Lip patterns have unique nature and individualism so can be used for human identification. The aim of this study was to find out the prevalence of Type II lip print patterns among medical students in a medical college.

**Methods:**

A descriptive cross-sectional study was conducted in a medical college from 5 March 2022 to 1 April 2022 after receiving ethical approval from the Institution Review Committee (Reference number: 057-078/079). The lip print pattern was categorized according to Tsuchihanshi's classification. Convenience sampling was done. Point estimate and 95% Confidence Interval were calculated.

**Results:**

Among 100 medical students, the Type II lip pattern was seen in 26 (26%) (17.40-34.60, 95% Confidence Interval) students. The largest number of participants was seen in 21 (80.76%) from the age group of 20-25 years.

**Conclusions:**

The prevalence of Type II lip pattern among medical students was similar to other studies done in similar settings.

## INTRODUCTION

The study of lip patterns, cheiloscopy, deals with the study of various furrows and grooves on the vermillion border.^1^ The pattern is unique and individualistic so used for identity fixation.^2^ Lip patterns help link a person at the scene of the crime. According to Locard's exchange principle, when two objects come into contact, there is always a transfer of material from one object to the other.^1-4^ Lipsticks that don't leave any visible trace after contact have been developed.^5^

Lip prints are characterised by their permanency and are referred to as persistent lip prints which can be lifted using materials such as aluminium powder and magnetic powder.^5^ There is a huge potential for Deoxyribonucleic acid (DNA) typing from lip print.^6^

The aim of this study was to find out the prevalence of Type II lip print patterns among medical students in a medical college.

## METHODS

This was a descriptive cross-sectional study carried out in Nepal Medical College and Teaching Hospital (NMCTH) for a duration of two months from 5 March 2022 to 1 April 2022. Ethical approval was obtained from the Institution Review Committee of NMCTH (Reference number: 057-078/079). After explaining the objective and plan of study and fulfilling inclusion and exclusion criteria, informed written consent was taken from the students. MBBS students in the age group of 18-25 years were included. Any kind of deformities in lips like trauma or scars in the lips, any active or passive lesions on the lip, or known hypersensitivity to lipsticks were excluded from the study. Convenience sampling was done. The sample size was calculated using the formula:


$$\begin{array}{ll} \text{n} & ={\text{Z}}^{2}\times \frac{\text{p}\times \text{q}}{{\text{e}}^{\text{2}}}\\ & =1.96^{2}\times \frac{0.278\times 0.722}{0.09^{2}}\\ & =96 \end{array}$$

Where,

n= minimum required sample size
Z= 1.96 at 95% Confidence Interval (CI)
p= prevalence of type II lip pattern, 27.8%^6^
q= 1-p
e= margin of error, 9%

Hence, the minimum required sample size was 96. However, a sample size of 100 was taken for the study. The lips of all the participants are cleaned as any remains of food or cosmetics can alter the actual shape and simply be evident as a stain. After properly cleaning the lips, dark-coloured lipstick was applied with a single stroke evenly on the lips. The subjects were then asked to rub both lips to evenly spread the applied lipstick. Over the lipstick, the glued portion of the cellophane tape strip was placed. The subjects are then asked to make a lip impression in the normal resting position of the lips by dabbing in the centre first and then pressing it uniformly towards the corner of the lips.^7^

The cellophane strip was then gently removed and stuck to the white chart paper for permanent record purposes and then was visualised by a magnifying lens. Further, proper lighting was focused to clearly make out the differences between the white and dark areas.^8^ The lip prints thus obtained were coded while noting the name and sex of the respective individuals. The number of lines and furrows present, their length, branching and combinations were noted. The sex of the individual is determined. All the lip prints obtained were studied and interpreted by the examiner to identify the sex of the subjects and the results were analyzed for the presence or absence of common lip print patterns in males and females.

Classification of the lip prints was done according to Tsuchihanshi's classification.^4^ The examiner studied and interpreted all the lip prints obtained to identify and analyse the common lip print patterns in males and females. The collected data were entered and analysed in IBM SPSS Statistics 16.0. Point estimate and 95% CI were calculated.

## RESULTS

**Figure 1:**
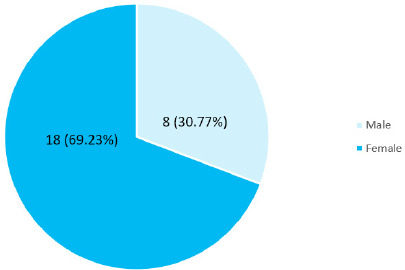
Genderwise distribution of Type II lip pattern (n= 26).

Among 100 students, the Type II lip pattern was seen in 26 (26%) (17.40-34.60, 95% CI) students. Among them, females were 18 (69.23%) (Figure 1).

The largest number of participants was seen in 21 (80.76%) from the age group of 20-25 years (Table 1).

**Table 1:** Agewise Distribution of Type II lips pattern (n= 26).

Age group	n (%)
20-24	21 (80.76)
25-29	4 (15.38)
30-34	1 (3.84)

## DISCUSSION

In our study Type II lip pattern is found in 26% among total study participants and is more predominant in females than males. This finding is inconsistent with the studies conducted by others^6,9-14^ but different from the study conducted in Andhra Pradesh.^15^ The finding of our study cannot be generalized as the study population from our study consists of participants from a single organization and has a very small sample size.

Our study concluded that even though all the lip prints belonged to one of the six categories none of the two sets of lip prints was identical to each other making each print individualistic of the other. This finding is in line with almost all the literature reviewed.^10,16-18^ Our study had age group from 20 to 35 age group a similar with various other studies that have had the study groups to be of the age ranges of between 21 to 35 years.^3,4,9,14,15,19,20^ According to another study, statistical analysis has shown there to be a significant difference in lip pattern between sexes.^21^

Even though lip prints have been an intriguing concept for many detectives there is still a need for systematization of the patterns by using standard methods.^22^ There have been reports illustrating the lip patterns to be consistent throughout the period of many years and not being subjected to any gross changes.^23^ Furthermore, studies have shown there to be no similarity among lip patterns even among siblings as with fingerprints.^22,23^ Furthermore, there is lacking literature regarding the occurrence of various patterns of Type II lip patterns and a study was done to look into the varieties of sub-varieties of Type II lip prints among males and females.^23^

Our study had some limitations. The small sample size and selection of participants from a single institution for study with a close age gap is the limitation of our study. Furthermore, the association between the study variables could not be made as this was a descriptive cross-sectional study.

## CONCLUSIONS

The prevalence of type II was similar to other studies done in similar settings. People's identification has always been of utmost importance in forensic medicine and hence, lip patterns can prove to be of as much importance and significance as fingerprints as a tool for human identification.
